# Advances on Bacterial and Fungal Biofilms for the Production of Added-Value Compounds

**DOI:** 10.3390/biology11081126

**Published:** 2022-07-27

**Authors:** Fábio M. Carvalho, Ana Azevedo, Marta M. Ferreira, Filipe J. M. Mergulhão, Luciana C. Gomes

**Affiliations:** 1 LEPABE—Laboratory for Process Engineering, Environment, Biotechnology and Energy, Faculty of Engineering, University of Porto, Rua Dr. Roberto Frias, 4200-465 Porto, Portugal; up201502963@edu.fe.up.pt (F.M.C.); acma@fe.up.pt (A.A.); up201705666@edu.fc.up.pt (M.M.F.); filipem@fe.up.pt (F.J.M.M.); 2 ALiCE—Associate Laboratory in Chemical Engineering, Faculty of Engineering, University of Porto, Rua Dr. Roberto Frias, 4200-465 Porto, Portugal

**Keywords:** productive biofilms, cell immobilization, biofilm reactor, recombinant protein, added-value product

## Abstract

**Simple Summary:**

The production of bio-based materials, including organic acids, antibiotics, enzymes, ethanol, and hydrogen, is generally done by the cultivation of suspended cells rather than using immobilized cells. However, several studies suggest the application of productive biofilms as a reliable alternative for biocatalysis, with many advantages over suspended-growth systems. This review gives an overview of the breakthrough in the application of biofilm platforms for the sustainable production of valuable compounds, with particular insight into the latest advances in the production of recombinant proteins. Productive biofilms are shown to improve production rates and product yields, demonstrating great potential for industrial applications.

**Abstract:**

In recent years, abundant research has been performed on biofilms for the production of compounds with biotechnological and industrial relevance. The use of biofilm platforms has been seen as a compelling approach to producing fine and bulk chemicals such as organic acids, alcohols, and solvents. However, the production of recombinant proteins using this system is still scarce. Biofilm reactors are known to have higher biomass density, operational stability, and potential for long-term operation than suspended cell reactors. In addition, there is an increasing demand to harness industrial and agricultural wastes and biorefinery residues to improve process sustainability and reduce production costs. The synthesis of recombinant proteins and other high-value compounds is mainly achieved using suspended cultures of bacteria, yeasts, and fungi. This review discusses the use of biofilm reactors for the production of recombinant proteins and other added-value compounds using bacteria and fungi.

## 1. Introduction

Biofilms are aggregates of microorganisms, such as bacteria, fungi, or algae, which are protected by a matrix of extracellular polymeric substances (EPS) that are usually attached to a solid surface that can be organic or inorganic [1,2]. Research in biofilms tends to focus on their detrimental effects on sectors such as health, food, and the maritime industry [3,4]. These effects range from persistent infections on medical devices [5], equipment clogging, heat transfer reduction and product degradation in the food industry [6], and the increment in frictional drag and consequent fuel consumption in marine vehicles [7]. The beneficial properties of biofilms include not only wastewater treatment [8], bioremediation, and removal of toxic pollutants [9,10], but also the production of added-value substances, such as organic acids, enzymes, alcohols, and recombinant proteins [11,12,13,14,15].

Recombinant proteins and other added-value compounds are being produced in biofilm reactors due to several advantages of this platform when compared to suspended cell systems. Biofilm reactors can (1) retain more biomass per unit volume, increasing production rates and yields, (2) resist stress conditions such as toxic compounds, (3) reduce the risk of washout (which eliminates the need for repeated inoculations during subsequent batch fermentation), and (4) reduce fermentation times and exhibit long-term activity [11,15,16,17]. However, some challenges need to be addressed, namely, (1) limitations of substrate and oxygen diffusion, which can increase population heterogeneity, (2) the complex maintenance of a pure culture in consecutive operations, (3) biofilm reactors are difficult to scale up [17], and (4) product secretion to the extracellular medium can be challenging, leading to difficulties in downstream processes [15].

Several studies propose the application of biofilms as robust, self-immobilized, and self-regenerating systems in the production of added-value chemicals and specific proteins [11,18,19]. Hence, this review intends to outline the advances in the production of recombinant proteins using biofilms, as well as to give an overview of the main added-value compounds produced using biofilms as a biocatalytic system.

## 2. Production of Added-Value Chemicals

**Figure 1:**
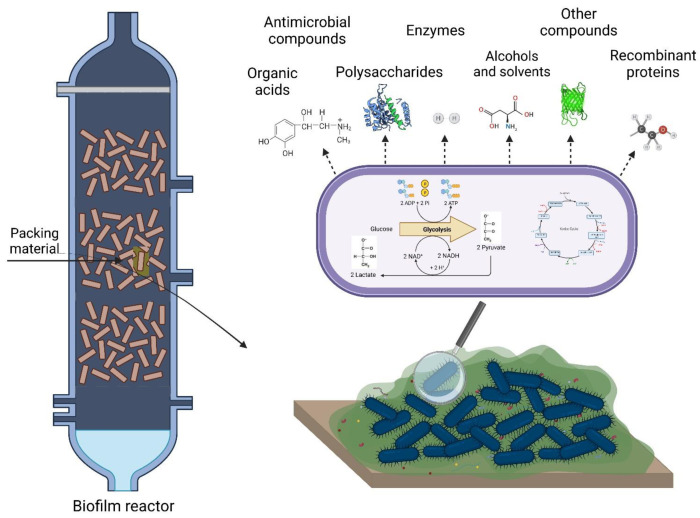
Added-value compounds produced in biofilm reactors.

In recent years, microbial biofilms have emerged as a new generation of biocatalysts due to their potential for the sustainable production of added-value chemicals [16,20,21], including organic acids, enzymes, polysaccharides, antimicrobial compounds, alcohols and solvents, and other products (Figure 1). This production typically resorts to a variety of biofilm reactors in which microorganisms attach to support materials [11,17,18,19]. The most common reactor types used to produce these substances are stirred-tank [22,23,24] and packed-bed reactors [25,26,27]. The packed-bed reactor is usually filled with densely packed solid supports, which provide high interfacial areas, whereas stirred-tank reactors integrate inserts and/or particles [19]. Additionally, membrane biofilm reactors with a porous gas-permeable membrane (e.g., silicone [28,29] and polysulphone [30]) are often used for these bioreactions [28,29]. Other configurations include fluidized-bed reactors [31,32], airlift reactors [33,34], bubble column reactors [35,36], rotating-disk reactors [37,38], or tubular biofilm reactors [39]. Thus, the choice of the reactor and feeding strategy (batch, fed-batch, and continuous mode) should be molded to the process conditions and nutritional requirements of the producing microorganisms.

Several support materials have been employed for the immobilization of microorganisms in high biomass concentrations inside the reactors. The supports must be prone to adhesion of microorganisms, be widely available and inexpensive, resist high mechanical forces, and be non-toxic [16,18,19]. Synthetic materials employed as supports in biofilm reactors may include ceramics [26,40], silicone [41,42], polyethylene [43,44,45], polyurethane [46,47], clay bricks [27], polypropylene [48], and glass [39]. Natural polymers, such as alginate [49,50] and carrageenan [22], and some lignocellulosic materials, such as cotton [51,52], have also been used to immobilize microbial cells. Many agriculture-based waste materials have been used to create biofilm supports, such as corn stalks [53] or charcoal pellets produced from waste mushroom medium [54]. A good example is the loofah sponge, an inexpensive and environmentally friendly support matrix obtained from the ripped dried fruit of *Luffa aegyptica* [55,56], applied to produce lactic and gibberellic acids. Furthermore, many studies extensively implemented a specific class of plastic composite supports (PCS) for biomass immobilization due to the channeling of agricultural wastes to produce valuable compounds [24,38,57,58,59,60]. PCS are a blend of polypropylene, nutritious agricultural materials (e.g., oat hulls, soybean flour, and cornstarch), microbial nutrients (e.g., yeast extract, and bovine albumin), and mineral salts [60,61,62], usually produced in the form of chips [57], rings/disks [60], or tubes fixed to the agitator shaft of stirred-tank biofilm reactors [63]. Hence, this support simultaneously provides attachment areas for biofilm development and nutrients for the growth and synthesis of products.

Since the production of chemicals and fuels through biocatalytic processes in biorefineries is strongly impacted by raw material costs [64], it is driven by the utilization of renewable feedstocks, low in cost, abundant, and readily available, to sustainably produce commercially valuable products [65]. These raw materials do not compete with food crops and often comprise industrial wastes such as whey and milk permeates [25,55], molasses [46], olive mill wastewater [26], potato starch [56], and rice straw [66]. Potato waste and rice straw hydrolysates were used by Izmirlioglu and Demirci [67] and Todhanakasem et al. [68], respectively, as fermentation media to benefit from available and inexpensive waste materials to make ethanol production more sustainable. In another study, a complex medium containing the liquid fraction of deacetylated corn stover hydrolysate was used as a substrate for succinic acid production [65]. Although renewable feedstock may be cost-effective, their commercial feasibility requires a compromise between material costs and fermentation productivities and yields. Since the nutrients might be less accessible to microbial consumption, sometimes an additional step is needed to make their carbohydrate fraction available for microbial conversion [69]. During this pre-treatment, inhibitory compounds are produced, which can decrease production rates and yields, demanding an extra step for the removal of these substances and increasing the process costs [70]. Biofilms can tolerate such hazardous environments more easily than suspended cells, conferring a great advantage in this case.

### 2.1. Organic Acids

The production of a wide variety of organic acids in biofilm reactors is very popular due to their higher robustness to changing environmental conditions, in particular, a decrease in pH [19]. The organic acids produced in these systems include lactic, succinic, acetic, citric, fumaric, gibberellic, glycolic, propionic, and kojic acids (Table 1). Ho and colleagues reported a few studies on the production of lactic acid (widely used in chemical, pharmaceutical, and food industries [71]) in biofilm reactors, studying the characteristics of PCS and their effects on biofilm formation and lactic acid production [72], and the effects of different agricultural components on the properties of PCS [73]. These supports stimulated biofilm formation and improved the productivity of lactic acid in repeated-batch fermentations up to 4.3 g·L^−1^·h^−1^ at a starting glucose concentration of 100 g·L^−1^ [62]. The immobilized cells shortened the total fermentation time up to 61% and increased the lactic acid productivity of *L. casei* up to 70% relative to suspended cells. Following this, the fungi *Rhizopus oryzae* was used by Tay and Yang [51] to produce lactic acid in a rotating fibrous bed bioreactor. Glucose and cornstarch were the fermentation substrates tested. The highest lactic acid productivity of 2.5 g·L^−1^·h^−1^ was obtained from glucose in fed-batch fermentation with a yield of 90%, whereas a lactic acid yield close to 100% was achieved with cornstarch, despite the lower productivity of 1.65 g·L^−1^·h^−1^. Moreover, the immobilization with the cotton cloth restrained control and operation problems in the reactor observed with freely suspended fungal cells. More recently, Cuny et al. [39] used *Lactobacillus delbrueckii* to produce lactic acid in a horizontal tubular biofilm reactor. This biofilm system was operated in continuous mode for 3 weeks under different flow velocities and demonstrated good stability. The productivity increased with the flow velocity since, at low flow velocities, the higher retention times cause a strong pH drop generated by lactic acid accumulation, inhibiting the growth rate and production. The maximum productivity obtained was 10 g·L^−1^·h^−1^ with a product yield of 94%. The biofilm system demonstrated superior cell density and productivity of lactic acid over a batch culture by a factor of 19 and 6–8, respectively.

Urbance et al. [63,74] reported two works on the production of succinic acid by *Actinobacillus succinogenes* using PCS for biofilm formation. In their first study, they developed a medium supporting the growth and succinic acid production by *A. succinogenes* and screened customized PCS blends for cell immobilization and succinic acid production [74]. Then, the effectiveness of these supports was evaluated in repeated-batch and continuous fermentation with immobilized and suspended-cell systems [63]. For the continuous mode in the PCS bioreactor, as the dilution rate increased, succinic acid final concentrations and percentage yields decreased while productivity increased. A maximum of 8.8 g·L^−1^·h^−1^ was reached at a dilution rate of 1.2 h^−1^, whereas a maximum productivity of 7.0 g·L^−1^·h^−1^ was obtained at a dilution rate of 1.0 h^−1^ for suspended culture. In batch fermentation, *A. succinogenes* was able to tolerate high initial glucose concentrations. However, the overall production rate was higher at lower glucose concentrations (0.9 g·L^−1^·h^−1^), which suggests the need to continuously remove the succinic acid from the fermentation broth due to product inhibition.Another series of studies exploring the continuous production of succinic acid was performed by Bradfield and colleagues [42,65,75]. In their last study, Bradfield and Nicol [42] employed different types of biofilm supports (tightly bound wooden sticks, silicone-tubing segments, and loosely spaced wooden sticks) in three separate fermentations using a xylose feed stream. The results showed succinic acid yields on xylose of 0.55–0.68 g·g _xylose_^−1^, titers of 10.9–29.4 g·L^−1,^ and productivities of 1.5–3.6 g·L^−1^·h^−1^ at different dilution rates. Although these levels were lower than the maximum achieved on glucose (4.4 g·L^−1^·h^−1^) in their previous work [75], the authors believe that succinic acid productions on xylose and glucose are comparable, suggesting that industrially relevant biomass feedstocks can be employed in the production of valuable compounds. Moreover, Ferone et al. [76] investigated the continuous anaerobic production of succinic acid by *A. succinogenes* for more than 5 months in a packed-bed biofilm reactor with Tygon rings as immobilization support. The bioreactor was fed with a synthetic medium simulating the composition of a lignocellulosic hydrolysate and carbon dioxide (CO_2_) for the succinic acid production pathway. The maximum succinic acid productivity (35 g·L^−1^·h^−1^) was obtained using glucose as a carbon source at a dilution rate of 1.9 h^−1^ and was the highest productivity reported so far using biofilms reactors. However, the optimum balance between succinic acid concentration, productivity, and sugar conversion was obtained at a dilution rate of 0.5 h^−1^ (43 g·L^−1^, 22 g·L^−1^·h^−1^, and 88% glucose conversion, respectively).

In addition to lactic acid, *R. oryzae* was employed by Cao et al. [77,78] in the production of fumaric acid from glucose in a rotating-disk biofilm reactor with polysulfone plastic disks mounted on a horizontal shaft. The authors created an integrated system of simultaneous/continuous production and recovery of fumaric acid by an adsorption column coupled to the reactor [78]. When *R. oryzae* produces fumaric acid, a decrease in the pH below a certain threshold may stop the fermentation. Therefore, adsorbent resins were used to remove the free acid and moderate the decrease in pH, thereby enhancing the fermentation rate and maintaining cell viability. As a result, this biofilm reactor reached a concentration of fumaric acid of 85 g·L^−1^, a yield of 91% (w/w), and maximum productivity of 4.25 g·L^−1^·h^−1^ within 20 h (compared to 72 h in the suspended-cell reactor). Conversely, in a stirred-tank fermentation, the productivity was 0.9 g·L^−1^·h^−1^, about 5 times lower than with biofilms. The same rotating-disk reactor was operated, supplementing the medium with CaCO_3_ to neutralize the pH, as an alternative to the adsorbent unit, and the fumaric acid productivity in the biofilm reactor was 3.78 g·L^−1^·h^−1^ within 24 h, about 5 times higher than with the stirred-tank fermenter, and the fermentation time was shortened by one-third [77]. The biofilm reactor was operated for 2 weeks without loss of biological activity.

A rotating-disk biofilm reactor was similarly used by Wang et al. [79] to produce citric acid by *Aspergillus niger* immobilized in polyurethane foam disks. The volumetric productivity obtained with the immobilized cell culture was 0.9 g·L^−1^·h^−1^ (weight yield of 72%), about 3 times higher than a stirred-tank fermenter with suspended cell culture (0.33 g·L^−1^·h^−1^; weight yield of 60%). Additionally, the immobilized biofilm was active for eight repeated-batch cycles without losing bioactivity. More recently, Yu et al. [80] developed a new carrier material termed PAF201 (polymeric porous foam made of polyurethane and carbon black) for *A. niger* immobilization with improved citric acid yield and productivity levels. PAF201 demonstrated improved cell immobilization and glucose consumption compared with other materials. Moreover, this carrier reduced the fermentation period (72 h) compared to planktonic cells (96 h). In a repeated fed-batch fermentation, the production of citric acid using cassava medium and immobilized *A. niger* showed maximum citric acid yields, concentrations, and productivity of 90%, 163 g·L^−1^, and 2.26 g·L^−1^·h^−1^, respectively, which were kept constant in all batches, demonstrating long-term stability. On the other side, the citric acid productivity of the suspended cell system was almost half of immobilized fermentation (1.41 g·L^−1^·h^−1^).

As for acetic acid, Horiuchi et al. [54] operated a packed-bed reactor with *Acetobacter pasteurianus* immobilized in charcoal pellets. The acetic acid productivity reached a maximum of 6.5 and 3.9 g·L^−1^·h^−1^ with a supply of O_2_-enriched air (40%) and normal aeration, respectively, indicating that the process was limited by oxygen transfer. The charcoal pellets were obtained at low cost from agricultural wastes and presented a porosity and specific surface area appropriate for bacterial adhesion; also promoting good operational stability since the system was continuously operated for 180 days. On the other side, Talabardon et al. [52] investigated the production of acetic acid from lactose and milk permeate, a by-product of the ultrafiltration of milk, using an anaerobic thermophilic co-culture of *Clostridium thermolacticum* and *Moorella thermoautotrophica*. In this fermentation process, *C. thermolacticum* converts lactose into lactic acid, which is thereby converted into acetic acid by *M. thermoautotrophica*. The fermentation kinetics were compared between a suspended cell reactor and an immobilized-cell fibrous-bed reactor in fed-batch fermentations at 58 °C. The acetic acid final concentration (22.0–22.5 g·L^−1^) and productivity (0.18–0.54 g·L^−1^·h^−1^) achieved in a fibrous-bed bioreactor using either lactose or milk permeate were significantly higher compared to those from the suspended cell fermentation (final concentration, 15 g·L^−1^; productivity, 0.06–0.08 g·L^−1^·h^−1^). The higher productivity of the fibrous-bed bioreactor was attributed to the higher cell density (20 g·L^−1^), approximately 10 times higher than in the planktonic culture (2 g·L^−1^). Additionally, the higher acetic acid yields and concentrations in the bioreactor were attributed to the mitigation of ethanol production as a by-product, and to the ability of the immobilized cells to adapt and tolerate higher product concentrations, respectively.

Gibberellic acid was produced from a milk permeate by the fungi *Fusarium moniliforme* immobilized in loofah sponge disks [55]. The effect of incubation temperature, initial pH, number of disks, and its reusability for gibberellic acid production was evaluated. The best gibberellic acid productivity of 15.6 mg·L^−1^·h^−1^ was reached at pH 5 after 6 days of incubation. Additionally, the *F. moniliforme* cells immobilized on the loofah sponge were reused in repeated batches and showed high production stability.

Liu et al. [59] used a PCS-immobilized bioreactor to produce kojic acid (an acid with strong metal chelating capacity widely used in cosmetic and food industries) by *Aspergillus oryzae* in repeated-batch fermentations. The use of a nitrogen-deficient (Ndef) medium created differences in mycelium morphology between the free suspension and the PCS-immobilized cultures. Mycelia in the Ndef medium had a feather-like structure, while in suspension, mycelia were more compact. These morphology changes were assumed to increase the surface area for absorbing more nutrients, which resulted in increased kojic acid production. In addition, RNA expression (*koj*A and *koj*T) under nitrogen starvation was 2.5 times higher than the control with full nitrogen, indicating that nitrogen deficiency influenced kojic acid production at the transcriptional level. This PCS immobilized fermentation system decreased the time needed to reach higher productions and productivities, where 83.47 g·L^−1^ of kojic acid was produced with a productivity of 3.09 g·L^−1^·day^−1^, which is higher than free-suspension in batch fermentation.

**Table 1:** Different classes of organic acids produced in biofilm reactors.

Product	Producers	Substrate	Immobilization Material	Reactor Type	Process Time (h)	Maximum Productivity (g·L^−1^·h^−1^)	Productivity Increment ^c^	Ref.
Lactic acid	*Lactobacillus casei* subsp. *rhamnosus*	Glucose as CS and YE as NS	PCS	Packed-bed reactor (B) ^b^	1584	4.3	1.5	[62,72,73]
				Stirred-tank reactor (C)	n.d.	9.88	n.a.	[24]
	*Lactobacillus delbrueckii*	Glucose as CS and YE as NS	Glass	Tubular biofilm reactor (C)	504	10	6–8	[39]
		MRS medium with molasses as CS	Polyurethane foam	Packed-bed biofilm reactor and stirred-tank reactor (C)	1000	5	4	[46]
	*Rhizopus oryzae*	Glucose and cornstarch as CS	Cotton cloth	Rotating fibrous bed bioreactor (FB)	200	2.5	n.a.	[51]
		Potato starch	Loofah sponge	Airlift reactor (B)	48	5 g·L^−1^	1.7	[56]
Succinic acid	*Actinobacillus succinogenes*	Xylose as CS and YE as NS	Wooden sticks and silicone-tubing segments	n.d. (C)	1500	3.6	n.a.	[42]
		Glucose as CS and YE as NS	PCS	Stirred-tank reactor (B, C) ^b^	n.d.	8.8	1.25	[63,74]
		Glucose and CO_2_ as CS, and YE as NS	Poraver beads	Packed-bed reactor (C)	80	10.8	n.a.	[81]
			Tygon rings		3600	35	n.a.	[76]
Fumaric acid	*Rhizopus oryzae*	Glucose as CS	Polysulfone plastic disks	Rotary biofilm contactor (FB) ^b^	20 ^a^	4.25	5	[77,78]
Citric acid	*Aspergillus niger*	Sucrose as CS	Polyurethane foam	Rotary biofilm contactor (FB) ^b^	120 ^a^	0.90	3	[79]
		Sucrose and sugar cane juice as CS	Cellulose microfibrils	Recycle reactor (C, FB)	624	2.08	1.8	[82]
		Glucose as CS dissolved in wheat bran extract and cassava-based medium	Polyurethane and carbon black foam	Flasks (FB) ^b^	72 ^a^	2.26	2	[80]
Acetic acid	*Acetobacter pasteurianus*	Glucose as CS and ethanol as BS	Charcoal pellets	Packed-bed reactor (C)	4320	6.45	n.a.	[54]
	*Clostridium thermolacticum* and *Moorella thermoautotrophica*	Lactose and milk permeate as CS and trypticase and YE as NS	Cotton towel overlaid with a stainless-steel wire cloth	Fibrous-bed bioreactor (B, FB) ^b^	336 ^a^	0.54	6	[52]
Propionic acid	*Propionibacterium acidipropionici*	Sorghum bagasse hemicellulosic hydrolysate	Sorghum bagasse	Glass column (B) ^b^	146	1.17	6	[83]
Glycolic acid	*Pseudomonas diminuta*	Ethylene glycol as the BS	Stainless steel structured packing	Aerated trickle-bed biofilm reactor (C)	1536	1.6	5	[84]
Gibberellic acid	*Fusarium moniliforme*	Milk permeate	Loofah sponge	Shaking flask (B) ^b^	144	1.6 × 10^−2^	1.4	[55]
Gluconic acid	*Aspergillus niger*	Deproteinized whey	Polyurethane foam	Erlenmeyer flasks (B)	72	92 g·L^−1^	1.33	[85]
Fatty acids (acetate, propionate, and butyrate)	Methanogens and acid-producing bacteria	Methane as BS	Hollow fiber membranes	Membrane biofilm reactor (B) ^b^	12 ^a^	0.42	n.a.	[86]
Kojic acid	*Aspergillus oryzae*	Glucose as CS	PCS	Shaking flasks (B) ^b^	648	0.13	>1	[59]

^a^ batch duration; ^b^ repeated-batch or fed-batch mode; ^c^ Productivity increment corresponds to the productivity ratio between biofilms and suspended cell processes. When productivity increment is not reported, it was calculated as the ratio between the maximum productivity obtained with biofilms and the maximum productivity obtained with planktonic cultures. Abbreviations: B, batch culture; C, continuous feeding; FB, fed-batch culture; CS, carbon source; YE, yeast extract; NS, nitrogen source; BS, biotransformation substrate; PCS, plastic composite supports; MRS, De Man, Rogosa, and Sharpe broth; n.a., not applicable; n.d., not described.

### 2.2. Enzymes

The production of enzymes by the application of biofilm reactors has been scarcely investigated (Table 2). The production of cellulase, a lignocellulosic material with applicability in biofuel production and textile, paper, and pulp industries [87], using biofilm reactors was firstly reported by Webb et al. [88] using *Trichoderma viride* immobilized on stainless steel particles in a spouted-bed fermenter. They obtained a volumetric productivity of 31.5 U·L^−1^·h^−1^, which was more than three times higher compared with suspended cells. Since then, a few studies were performed on cellulase production in biofilms. Hui et al. [89] examined the stability of the *Aspergillus terreus* suspended cells and immobilized onto woven nylon pads with respect to cellulase production under repeated-batch fermentations. They found that the immobilization extended enzyme production for longer periods (about 120 days vs. 40 days) with a nearly 4.5-fold increase in productivity (with a cumulative enzyme activity of 453 U compared to 114 U) when compared to suspended cells.

Other ligninolytic enzymes, lignin (LiP) and manganese (MnG) peroxidases, were produced by the white-rot fungus *Phanerochaete chrysosporium*. Solomon and Petersen [30] described the production of these ligninolytic enzymes in a polysulfone membrane gradostat bioreactor. The study of the effect of operating parameters on enzyme production revealed higher activities at higher temperatures and lower glucose and ammonium concentrations. The maximum LiP and MnP were 35 and 96 U·L^−1^, respectively. The same biofilm system was used by Govender et al. [90] for the continuous production of MnP. In an initial screening, the authors optimized the effect of nutrient additives (Mn^2+^, Tween 80, and soybean-derived phospholipids) and oxygenation on MnP production and biofilm morphology and physiology. Oxygenation tangential to the biofilm has shown higher peroxidase activity (112 U·L^−1^) compared with oxygenation via a side arm (39 U·L^−1^) and bubbling O_2_ into the media (66 U·L^−1^). Additionally, the nutrient additives enhanced MnP activity both individually and when combined, resulting in a 58% increase in peroxidase activity compared to the conventional medium and a productivity of 1.3 U·L^−1^·h^−1^ under optimal conditions. In addition, Khiyami et al. [23] investigated the production of LiP and MnP in a biofilm stirred tank reactor holding PCS tubes. The addition of veratryl alcohol, a production activator, and aeration effectively improved the yield. The highest LiP and MnP activities were 50 and 63 U·L^−1^, respectively.

Yang et al. [91] described the application of *Rhizopus arrhizus* immobilized in polyurethane for lipase production. Lipase production was optimized regarding culture conditions where temperatures under 27 °C, a neutral pH, increasing levels of aeration, and the use of soybean flour and oils as nitrogen and carbon sources, respectively, enhanced lipase production and activity. The lipase productivity of immobilized cells during the repeated-batch fermentation in 250 mL flasks (17.6 U·mL^−1^·h^−1^) was about three times higher than a 5 L fermentor (6.1 U·mL^−1^·h^−1^), and the fermentation time was also shortened (nine and six consecutive batches in 140 h, respectively). This demonstrates the difficulty in reproducing the lab-scale results in large-scale biofilm reactors.

**Table 2:** Different classes of enzymes produced in biofilm reactors.

Product	Producers	Substrate	Immobilization Material	Reactor Type	Process Time (h)	Maximum Productivity (U·L^−1^)	Productivity Increment ^c^	Ref.
Cellulase	*Trichoderma viride*	Glucose as CS	Stainless steel spheres	Spouted-bed reactor (C)	336	31.5 U·L^−1^·h^−1^	3	[88]
	*Aspergillus niger*	Ground rice straw	Celite and polyurethane foams	Bubble column fermenter and shaking flasks (B)	168	1400	2	[66]
	*Aspergillus terreus*	Cellulose as CS	Woven nylon pads	n.d. (B) ^b^	2880	2400	4.5	[89]
Lignin peroxidase and Manganese peroxidase	*Phanerochaete chrysosporium*	Glucose as CS	Polysulfone capillary membrane	Membrane gradostat bioreactor (C)	336	LiP = 35 MnP = 96	n.a.	[30]
			PCS	Stirred-tank reactor (B) ^b^	144 ^a^	LiP = 50 MnP = 63	n.a.	[23]
			Polystyrene foam	Shaking flasks (B)	192	MnP = 421	1.2	[92]
		Phospholipid-rich medium	Polysulfone capillary membrane	Membrane gradostat bioreactor (C)	552	1.3 U·L^−1^·h^−1^	n.a.	[90]
Lipase	*Rhizopus arrhizus*	Peanut oil as CS and soybean flour as NS	Polyurethane	Shaking flasks (B) ^b^	140	1.76 × 10^4^ U·L^−1^·h^−1^	n.a.	[91]

^a^ batch duration; ^b^ repeated-batch or fed-batch mode; ^c^ Productivity increment corresponds to the productivity ratio between biofilms and suspended cell processes. When productivity increment is not reported, it was calculated as the ratio between the maximum productivity obtained with biofilms and the maximum productivity obtained with planktonic cultures. Abbreviations: B, batch culture; C, continuous feeding; SC, semi-continuous feeding; CS, carbon source; PCS, plastic composite supports; LiP, Lignin peroxidase; MnP, Manganese peroxidase; U, activity unit; n.a., not applicable; n.d., not described.

### 2.3. Polysaccharides

Compared to the other substances, the production of polysaccharides using biofilm reactors has barely been studied (Table 3). Bacterial cellulose was successfully produced by Cheng et al. [93,94] using *Acetobacter xylinum* immobilized in a PCS biofilm reactor. The high biomass density accumulated on the PCS resulted in a bacterial cellulose production of 7.05 g·L^−1^, about 2.5-fold higher than with the suspended-growth reactors (2.82 g·L^−1^) [94]. Moreover, improved mechanical properties (elastic deformation, strain at break, and mechanical strength) and thermal stability were observed for the PCS-grown bacterial cellulose. Higher production values were obtained more recently by Rahman et al. [95] which, similarly to Meleigy and Khalaf [55], used a natural loofah sponge as a scaffold for cell immobilization, in this case, using *Gluconacetobacter kombuchae* for the production of bacterial cellulose for 15 days. Bacterial cellulose production was compared between immobilized and non-immobilized cells, where immobilization on loofah supports resulted in approximately two times more product than in the absence of support. Moreover, several cultivation parameters were analyzed and optimized, including the initial pH, static or shaking conditions, inoculum size, nitrogen source, carbon/nitrogen ratio, and supplements that facilitate cellulose production (ethanol and acetic acid). A maximum cellulose production of 24 g·L^−1^ was obtained under shaking conditions, at an initial pH of 5.5, using yeast extract as a nitrogen source and a C/N ratio of 40 supplemented with ethanol.

Likewise, pullulan production was extensively investigated by Cheng et al. using *Aureobasidium pullulans* immobilized in PCS tubes connected to a stirred-tank reactor [58,96,97,98]. First, they tested numerous types of PCS with different compositions and assessed the effects of various pH profiles on pullulan production and biofilm formation [58]. A pullulan concentration of 32.9 g·L^−1^ with a purity of 96% was achieved in the biofilm reactor, which was 1.8 times higher than in a cell suspension, although the production rate was lower (0.44 g·L^−1^·h^−1^ vs. 0.68 g·L^−1^·h^−1^, respectively). Subsequently, they optimized the concentrations of sucrose and nitrogen sources (ammonium sulfate and yeast extract) in the medium for pullulan production using a Response Surface Methodology [96]. Medium optimization improved pullulan production up to 60.7 g·L^−1^ in 7 days, which was 2.4-fold higher than suspensions. Lastly, the effects of different concentrations of ammonium sulfate and sucrose and dilution rates were evaluated for continuous pullulan production [98]. The maximum pullulan production rate was improved compared with their previous studies (1.33 g·L^−1^·h^−1^ at a dilution rate of 0.16 h^−1^).

Additionally, Mesquita et al. [99] studied the production of xanthan gum with *Xanthomonas campestris* immobilized in polyurethane, and evaluated the storage stability and capacity for recycling the immobilized cells. The volumetric xanthan productivity with immobilized cells (0.62 g·L^−1^·h^−1^) was higher than in suspended-growth culture (0.12 g·L^−1^·h^−1^), indicating that immobilization improved the production of xanthan gum. Additionally, the immobilized cells demonstrated the capacity to be reused up to six times without losing significant activity. In a more recent study, Nejadmansouri et al. [100] compared the production of xanthan gum on different types of supports, demonstrating the improvement in xanthan production compared with the control without supports.

**Table 3:** Different classes of polysaccharides produced in biofilm reactors.

Product	Producers	Substrate	Immobilization Material	Reactor Type	Process Time (h)	Maximum Productivity (g·L^−1^·h^−1^)	Productivity Increment ^b^	Ref.
Bacterial cellulose	*Acetobacter xylinum*	Corn steep liquor with fructose as CS	PCS	Stirred-tank reactor (B)	120	5.9 × 10^−2^	2.5	[93,94]
	*Gluconacetobacter kombuchae*	Sucrose as CS and YE as NS	Loofah sponge	Shaking flasks (B)	360	6.7 × 10^−2^	2	[95]
	*Gluconacetobacter xylinum*	Corn steep liquor with fructose	PCS	Rotating-disk bioreactor (B) ^a^	120	1.0 × 10^−2^	n.a.	[38]
Pullulan	*Aureobasidium pullulans*	Sucrose as CS, ammonium sulfate and YE as NS	PCS	Stirred-tank reactor (B, C, FB)	168	1.33	3	[58,96,97,98]
Xanthan gum	*Xanthomonas campestris*	YM medium with sucrose as CS	Polyurethane	Shaking flask (B)	96	0.62	3.6	[99]
		YPD broth	Polyethylene	n.d. (B)	72	8 g·L^−1^	2.5	[100]

^a^ repeated-batch or fed-batch mode; ^b^ Productivity increment corresponds to the productivity ratio between biofilms and suspended cell processes. When productivity increment is not reported, it was calculated as the ratio between the maximum productivity obtained with biofilms and the maximum productivity obtained with planktonic cultures. Abbreviations: B, batch culture; C, continuous feeding; FB, fed-batch culture; CS, carbon source; YE, yeast extract; NS, nitrogen source; PCS, plastic composite supports; n.a., not applicable; n.d., not described.

### 2.4. Antimicrobial Compounds

Antibiotic production is usually performed using suspended-cell cultures [49]. However, cell immobilization proved to be efficacious and enhanced the productivity of antibiotics (including neomycin and cephalosporin) and other antimicrobial compounds such as bacteriocins and other proteins with bactericidal activity (Table 4) [101]. *Pediococcus acidilactici* immobilized on κ-carrageenan/locust bean gum gel beads was explored by Naghmouch et al. [102] for pediocin production in MRS broth and supplemented whey permeate medium. Pediocin is a bacteriocin with inhibitory action against some food-borne pathogenic and spoilage microorganisms involved in foodborne outbreaks [103]. An increased pediocin volumetric productivity was obtained in a repeated-cycle batch with immobilized cells (5461 AU·mL^−1^·h^−1^) compared with free cells (342 AU·mL^−1^·h^−1^). Moreover, the maximum activity of pediocin was reached after 0.75- and 2-h incubation cycles in MRS broth and whey permeate medium (2048 AU·mL^−1^·h^−1^), respectively, indicating the feasibility of using a low-cost medium such as whey permeate for high pediocin production.

Liu et al. [104] used a similar reactor type and immobilization supports for nisin production (a biopreservative for the food industry [105]) by *Lactococcus lactis*. Laboratory media (5.2 × 10^7^ AU·L^−1^·h^−1^) and whey permeate (1.0 × 10^7^ AU·L^−1^·h^−1^) originated similar productivities, which introduced whey permeate as an economical alternative for sustainable production of bacteriocins. Furthermore, the bioreactor was continuously operated for 6 months without clogging or contaminations, indicating long-term stability. Pongtharangkul and Demirci [106,107,108,109] also performed a set of studies on nisin production using a biofilm reactor with PCS tubes immobilizing *L. lactis*. The high biomass density attained with biofilm reactors was reflected in a shorter lag time of nisin production in comparison to the suspended-cell reactor, and sucrose (1100 IU·mL^−1^) increased nisin production substantially by 1.9-fold as related to glucose (579 IU·mL^−1^); however, high concentrations of sucrose stimulated lactic acid production, negatively affecting nisin production, as well as high magnesium concentrations [106]. Additionally, the levels of nisin production were greatly affected by the pH, andproduction in the biofilm reactor (3553 IU·mL^−1^) was about 1.8 times higher than in the suspended-cell system (2018 IU·mL^−1^) [107]. In a fed-batch fermentation, nisin production was enhanced for both suspended-cell (4188 IU·mL^−1^) and biofilm (4314 IU·mL^−1^) reactors, achieving 1.8- and 2.3-fold higher nisin titers than their respective batch fermentation due to the mitigation of substrate limitation and product inhibition [108]. Lastly, the implementation of an online recovery unit of silicic acid (adsorbent) coupled with a micro-filter module successfully recovered nisin from the fermentation broth and significantly improved nisin production (7445 IU·mL^−1^), approximately 4-fold when compared with the batch fermentation without the online recovery (1897 IU·mL^−1^) [109].

Srivastava and Kundu [34] produced Cephalosporin-C using *Cephalosporium acremonium* immobilized on an inert porous Siran carrier in an airlift reactor. Cephalosporin-C productivity was significantly improved in biofilm reactors (7.1 × 10^−3^ g·L^−1^·h^−1^) compared to suspended cell cultures (4.3 × 10^−3^ g·L^−1^·h^−1^). By using a similar reactor, Srinivasulu et al. [49] immobilized *Streptomyces marinensis* in alginate beads to produce neomycin, and also compared the effect of dilution rate and the use of planktonic cells on volumetric productivity. The maximum neomycin productivity with immobilized cells was 7.5 × 10^−3^ g·L^−1^·h^−1^ at a dilution rate of 0.065 h^−1^, about 2.5 times higher than with suspended cells.

More recently, Ercan and Demirci [110,111,112,113] performed stepped studies on the production of human lysozyme using the fungi *Kluyveromyces lactis* on PCS-grid biofilm reactors. Lysozyme is a lytic enzyme targeting bacterial cell walls with application in medicine, cosmetics, and food industries. Firstly, the growth conditions of *K. lactis* and the fermentation medium were optimized to maximize lysozyme production and biofilm formation on PCS [110,111]. The optimum conditions for lysozyme and biomass productions were different, so a shift in pH and aeration was done after biofilm formation to increase lysozyme secretion, achieving a lysozyme production of 173 U·mL^−1^. Later, the authors conducted fed-batch and continuous fermentations under the optimum operation conditions determined above [112]. Regarding the fed-batch fermentation, an initial feeding of glucose and continuous addition of lactose showed the highest lysozyme concentration and productivity (187 U·mL^−1^ and 5.9 U·mL^−1^·h^−1^, respectively) compared to their previous study in batch conditions (173 U·mL^−1^ and 4 U·mL^−1^·h^−1^). Continuous fermentation also supported significantly higher productivity (7.5 U·mL^−1^·h^−1^) over batch and fed-batch fermentations in a biofilm reactor and suspended cell reactor (4 U·mL^−1^·h^−1^). Finally, fermentation in a biofilm reactor was coupled to an online recovery system using silicic acid as an adsorbent to enhance lysozyme production and recovery [113]. The adsorption and desorption conditions of the recovery system were optimized, accomplishing 96% lysozyme adsorption and 98% desorption. The simultaneous fermentation and online lysozyme recovery improved the production to 280 U·mL^−1^, which was 63% higher than without the online recovery system (173 U·mL^−1^), demonstrating, just as Pongtharangkul and Demirci [109] did, that the use of recovery systems to recuperate bioactive compounds during fermentation has great potential to enhance the effectiveness of these processes.

**Table 4:** Different classes of antimicrobial compounds produced in biofilm reactors.

Product	Producers	Substrate	Immobilization Material	Reactor Type	Process Time (h)	Maximum Productivity	Productivity Increment ^c^	Ref.
Nisin	*Lactococcus lactis* subsp. *lactis*	Whey permeate	k-carrageenan/locust bean gum gel beads	Stirred-tank reactor (B) ^b^	1 ^a^	5.7 × 10^6^ AU·L^−1^·h^−1^	6.7	[22]
		Lactose and whey permeate as CS	Spiral wound fibrous matrix	Packed-bed reactor (C)	4320	5.2 × 10^7^ AU·L^−1^·h^−1^	n.a.	[104]
		Sucrose as CS	PCS	Stirred-tank reactor (B, FB) ^b^	12	7.6 × 10^6^ IU·L^−1^·h^−1^	1.8	[106,107,108,109]
Pediocin	*Pediococcus acidilactici*	MRS medium	Spiral wound fibrous matrix	Packed-bed biofilm reactor (C)	2160	4.2 × 10^5^ AU·L^−1^·h^−1^	n.a.	[114]
		MRS medium and supplemented whey permeate medium	k-carrageenan/locust bean gum gel beads	Stirred-tank reactor (B) ^b^	0.75 ^a^	5.5 × 10^6^ AU·L^−1^·h^−1^	16	[102]
Cephalosporin-C	*Cephalosporium acremonium*	Sucrose as CS	Siran beads	Airlift reactor (FB)	180	7.1 × 10^−3^ g·L^−1^·h^−1^	1.65	[34]
Neomycin	*Streptomyces marinensis*	Maltose as CS	Alginate beads	Airlift reactor (C)	16	7.5 × 10^−3^ g·L^−1^·h^−1^	2.5	[49]
				Erlenmeyer flasks	96	6.7 × 10^−2^ g·L^−1^·h^−1^	1.3	[115]
Lysozyme	*Kluyveromyces lactis*	Lactose as CS	PCS	Stirred-tank reactor (B, C, FB)	74	2.8 × 10^5^ U·L^−1^	1.8	[110,111,112,113]

^a^ batch duration; ^b^ repeated-batch or fed-batch mode; ^c^ Productivity increment corresponds to the productivity ratio between biofilms and suspended cell processes. When productivity increment is not reported, it was calculated as the ratio between the maximum productivity obtained with biofilms and the maximum productivity obtained with planktonic cultures. Abbreviations: B, batch culture; C, continuous feeding; FB, fed-batch culture; CS, carbon source; NS, nitrogen source; PCS, plastic composite support; MRS, De Man, Rogosa, and Sharpe broth; U, activity unit; AU, Anson unit; IU, international unit; n.a., not applicable.

### 2.5. Alcohols and Solvents

The production of alcohols and solvents, such as ethanol, butanol, and acetone, is a classic example of the use of biofilm reactors in the biotechnological scope (Table 5). Ethanol production was largely studied in different geometries of biofilm reactors. Kunduru and Pometto [116] investigated the continuous production of ethanol in a packed-bed reactor with PCS chips carrying *Zymomonas mobilis* or *Saccharomyces cerevisiae* in a long-term fermentation for 60 days. A maximum volumetric ethanol productivity of 536 and 76 g·L^−1^·h^−1^ were obtained for *Z. mobilis* and *S. cerevisiae* at dilution rates of 15 and 3 h^−1^, respectively, and these values were 100- and 15-fold higher than those obtained in suspension cultures. Later, lower productivity values of 2.31 g·L^−1^·h^−1^ were obtained by Izmirlioglu and Demirci [67] in a biofilm reactor with PCS-grid tubes immobilizing *S. cerevisiae*. The optimal growth parameters for *S. cerevisiae* in this biofilm reactor were found to be 34 °C, pH 4.2, and 100 rpm, reaching an ethanol concentration of 37 g·L^−1^ and a theoretical yield of 92%. The high porosity of PCS increased the surface area and established a very dense biofilm. In a different study, Shen et al. [117] surpassed the mass transfer limitations commonly observed in ethanol production by syngas fermentation through the use of a horizontal rotating packed bed (h-RPB) reactor. Biofilms of *Clostridium carboxidivorans* were immobilized on high-density polyethylene carriers and contacted the liquid and headspace alternately by the continuous reactor rotation. The gas transfer was more prominent in the headspace phase of the h-RPB reactor, which contributed significantly to cell growth and ethanol production. The reactor was continuously operated for 190 days at various rotational speeds, headspace pressures, and dilution rates. The maximum ethanol titer and productivity were 7.0 g·L^−1^ and 6.7 g·L^−1^·day^−1^, respectively, achieved at a dilution rate of 0.96 day^−1^, which was about 3.3-fold higher than those obtained in continuous stirred-tank reactors. The combination of a simple mechanical design, inexpensive parts for assembly, low power, and high ethanol demands make this reactor system efficient for syngas fermentation.

Gross et al. [29] used two recombinant *Pseudomonas* sp. strains (*Pseudomonas* sp. strain VLB120 pBT10 and *P. putida* PpS81 pBT10) in a silicone membrane biofilm reactor to continuously produce 1-octanol from octane. The volumetric productivities of both biofilms were 0.74 and 1.3 g·L^−1^·day^−1^ for about 30 and 7 days, respectively, similarly to the suspended cell reactor (1.0 g·L^−1^·day^−1^). Bioreactor aeration enhanced octanol synthesis by *P. putida* and decreased synthesis by *Pseudomonas* sp. strain VLB120, possibly due to the metabolization of octanol by the host’s alcohol dehydrogenases.

More recently, Hoschek et al. [118] used a dual-species biofilm of cyanobacterium *Synechocystis* sp. and *Pseudomonas taiwanensis*, both carrying the recombinant cyclohexane monooxygenase responsible for the oxyfunctionalization of cyclohexane to cyclohexanol. Their complementary properties regarding O_2_ metabolism resulted in higher cell densities compared to single-species biofilms since *P. taiwanensis* consumed the O_2_ fed to the capillary reactor, avoiding the inhibition of the *Synechocystis* sp. growth. This cooperation enabled the continuous cyclohexane conversion in cyclohexanol for a month with a productivity of 0.2 g·L^−1^·h^−1^.

The production of solvents (acetone, butanol, and ethanol—ABE) by solventogenic Clostridia (e.g., *Clostridium acetobutylicum* and *Clostridium beijerinckii*) fermentation has been attempted using microbial biofilms in order to make ABE production environmentally favorable by the use of renewable resources such as corn derivates, whey permeates, or different molasses [119,120]. Lee et al. [121] investigated the production of butanol by suspended or polyvinyl alcohol-immobilized cultures of *C. beijerinckii* in batch and continuous fermentations. The ratio of acetone/butanol was affected by the addition of acetate and butyrate, which enhanced the production of solvents, presumably due to a shift in the metabolic pathway toward solvent production. The addition of butyrate significantly increased butanol production in both immobilized and freely suspended cells. During continuous mode, the butanol productivity and yield were 0.40 g·L^−1^·h^−1^ and 0.44 g_-butanol_·g_-glucose_^−1^, respectively, about 2 times higher than those obtained with suspended cells (0.22 g·L^−1^·h^−1^ and 0.24 g_-butanol_·g_-glucose_^−1^). Moreover, supplementation with butyrate shifted the acetone/butanol ratio to 1:3 and prevented strain degeneration for 150 days, even in the presence of high butanol concentrations. In turn, Napoli et al. [122] used *C. acetobutylicum* immobilized in Tygon rings loaded in a packed-bed reactor for continuous butanol production for 750 h under several operational conditions (dilution rates, pH, and substrate concentrations). A complex media supplemented with lactose and yeast extract was employed to reproduce the nutritional characteristics of cheese whey wastewater. The maximum butanol productivity was 4.4 g·L^−1^·h^−1^ at a dilution rate of 1.0 h^−1^. Ethanol and acetone were also produced at lower concentrations alongside butanol (butanol selectivity of 88%). In addition, the existence of pH gradients towards the bottom layers of the biofilms was demonstrated, requiring a pH in the bulk higher than the optimal pH for suspended cell processes.

**Table 5:** Different classes of alcohols and solvents produced in biofilm reactors.

Product	Producers	Substrate	Immobilization Material	Reactor Type	Process Time (h)	Maximum Productivity (g·L^−1^·h^−1^)	Productivity Increment ^c^	Ref.
Ethanol	*Zymononas mobilis*	Glucose as CS and YE as NS	PCS	Packed-bed reactor (C)	1440	536	100	[116]
		Rice straw hydrolysate	Plastic and corn silk composites carriers	Packed-bed reactor (B, C) ^b^	120	Y_P/S_ = 0.47 g·g^−1^	n.a.	[68]
	*Saccharomyces cerevisiae*	Starch	Loofah sponge	Packed-bed reactor (B) ^b^	168 ^a^	0.25	1	[123]
		Potato waste hydrolysate	PCS	Stirred-tank reactor (B) ^b^	48	2.31	n.a.	[67]
	*Clostridium carboxidivorans*	Fructose as CS and syngas as BS	AnoxKaldnes K1 carriers	Horizontal rotating packed-bed reactor (C)	4560	0.28	n.a.	[117]
1-Octanol	Recombinant *Pseudomonas putida*	Octane as BS	Silicone membrane	Biofilm membrane reactor (C)	720	5.0 × 10^−2^	1.3	[29]
Cyclohexanol	*Synechocystis* sp. and *Pseudomonas taiwanensis*	Cyclohexane as BS	Glass	Capillary reactor (C)	720	0.2	n.a.	[118]
1,3-propanediol	*Klebsiella pneumoniae*	Glycerol as CS	Porous hydrophobic polyurethane	Fixed-bed reactor (FB) ^b^	1460	1.7	1.1	[47]
ABE solvents (acetone, butanol, and ethanol)	*Clostridia beijerinckii*	Glucose as CS and YE as NS	Corn stalk pieces	Biofilm reactor (C)	480	5.06	23	[53]
	*Clostridium acetobutylicum*	Lactose as CS and yeast extract as NS	Tygon rings	Packed-bed biofilm reactor (C)	750	4.4	n.a.	[122]

^a^ batch duration; ^b^ repeated-batch or fed-batch mode; ^c^ Productivity increment corresponds to the productivity ratio between biofilms and suspended cell processes. When productivity increment is not reported, it was calculated as the ratio between the maximum productivity obtained with biofilms and the maximum productivity obtained with planktonic cultures. Abbreviations: B, batch culture; C, continuous feeding; FB, fed-batch culture; CS, carbon source; YE, yeast extract; NS, nitrogen source; BS, biotransformation substrate; PCS, plastic composite support; Y_P/S_, ethanol yield; n.a., not applicable.

### 2.6. Other Compounds

Apart from organic acids, enzymes, polysaccharides, alcohols, and antimicrobial substances, other chemicals such as hydrogen, (S)-styrene oxide, benzaldehyde, and dihydroxyacetone have been produced using biofilm reactors (Table 6). In similar studies, Manssouri et al. [44] and Inoue et al. [43] produced hydrogen from sucrose-based synthetic wastewater in stirred anaerobic sequencing batch biofilm reactors by an anaerobic sludge immobilized on low-density polyethylene pellets. Using different feeding strategies, maximum molar hydrogen productivities of 39.9 mol·m^−3^·day^−1^ (batch) and 81.2 mol·m^−3^·day^−1^ (fed-batch) were obtained, respectively. More recently, Kongjan et al. [124] compared the application of a granule up-flow anaerobic sludge blanket reactor and an up-flow anaerobic packed-bed reactor with plastic biofilm supports for the continuous production of hydrogen by using a microbial consortium composed of moderate thermophilic cultures. The H_2_ production rate and yield at the optimal cultivation conditions were higher for the granules reactor (0.63 L-H_2_·L^−1^·h^−1^ and 0.25 L-H_2_·g_-xylose_^−1^, respectively) compared with the biofilm reactor (0.55 L-H_2_·L^−1^·h^−1^ and 0.22 L-H_2_·g_-xylose_^−1^, respectively), with acetate and butyrate as the main metabolite products. However, the maximum H_2_ production rate of 0.81 L-H_2_·L^−1^·h^−1^ was achieved by the biofilm reactor, though the H_2_ yield was lower (0.16 L-H_2_·g_-xylose_^−1^). Lower production rate values were obtained by Renaudie et al. [125] using a continuous hollow fiber liquid/gas membrane bioreactor originally seeded with sludge from a wastewater treatment plant containing *C. beijerinckii*, *Clostridium pasteurianum,* and *Enterobacter* sp. A maximum hydrogen productivity of 0.26 L-H_2_·L^−1^·h^−1^ was achieved, with acetate and butyrate being the main metabolite products from the glucose feed.

Furthermore, some studies reported continuous (S)-styrene oxide production through the epoxidation of styrene using the engineered *Pseudomonas* sp. strain VLB120DC as a biocatalyst attached in biofilm membrane reactors. Gross et al. [126] reached a maximum (S)-styrene oxide volumetric productivity of 70 g·L^−1^·day^−1^ using a tubular membrane reactor with a silicone membrane. This process was conducted for more than 50 days with no substrate or product mass transfer limitations, although high biomass concentrations introduced diffusional limitations of oxygen. On the other side, Halan et al. [40] employed a membrane biofilm reactor equipped with a microporous central ceramic unit for aeration and cell attachment, and obtained a maximum (S)-styrene oxide productivity of 28 g·L^−1^·day^−1^.

Additionally, the production of dihydroxyacetone (DHA) by *Gluconobacter oxydans* immobilized on silicone-coated Ralu-rings was investigated by Hekmat et al. [41] using a packed-bed bubble column reactor. Although the DHA yield from glycerol fermentation with and without cell immobilization was similar (0.87 and 0.85 kg·kg^−1^, respectively), DHA productivity was improved from 3.7 kg·m^−3^·h^−1^ using suspended biomass to 5.9 kg·m^−3^·h^−1^ with immobilized cells. The silicone matrix was demonstrated to be biocompatible, durable, mechanically stable, and have high oxygen permeability.

More recently, Roukas [48] attempted the production of carotene by the fungus *Blakeslea trispora* in a modified rotary biofilm reactor (MRBR) with polypropylene disks mounted on a polypropylene shaft. The MRBR enhanced the carotene production six times at optimal conditions compared with the conventional stirred-tank reactor (57.5 and 9.4 mg·L^−1^·day^−1^).

**Table 6:** Different classes of other added-value compounds produced in biofilm reactors.

Product	Producers	Substrate	Immobilization Material	Reactor Type	Process Time (h)	Maximum Productivity (g·L^−1^·h^−1^)	Productivity Increment ^c^	Ref.
Hydrogen	Anaerobic sludge	Sucrose-based synthetic wastewater	Low-density polyethylene	Stirred anaerobic sequencing batch biofilm reactor (FB, B) ^b^	2 ^a^	3.4 × 10^−3^ mol-_H2_·L^−1^·h^−1^	n.a.	[43,44]
			High-density polyethylene	Packed-bed reactor (C)	336–504	0.12 L-_H2_·L^−1^·h^−1^	n.a.	[45]
	Species of *Thermoanaerobacterium*	Xylose as CS	Plastic carriers	Up-flow anaerobic packed-bed reactor (C)	1368	0.81 L-_H2_·L^−1^·h^−1^	n.a.	[124]
	Activated sludge	Glucose as CS	Hollow-fiber membrane module of polytetrafluoroethylene	Liquid/gas membrane bioreactor (C)	92	0.26 L-_H2_·L^−1^·h^−1^	n.a.	[125]
Polyhydroxyalkanoates	*Bacillus* sp.	Mineral salt media with date syrup	PCS	Stirred-tank reactor (B) ^b^	30 ^a^	0.195	1.4	[127]
	Mixed microbial cultures	Acetic acid and fermented greenhouse residues	Biofilm carriers	Reactor tank	5400	35 mg·g _substrate_^−1^·h^−1^	n.a.	[128]
(S)-Styrene oxide	*Pseudomonas* sp. strain VLB120ΔC	Glucose as CS and styrene as BS	Silicone membrane	Tubular membrane reactor (C)	1200	2.92	n.a.	[126]
		Styrene as BS	Microporous ceramic aeration unit	Biofilm membrane reactor (C)	720	1.17	n.a.	[40]
Dihydroxyacetone	*Gluconobacter oxydans*	Glycerol as CS and YE as NS	Silicone-coated Ralu rings	Packed-bed bubble column reactor (FB) ^b^	432	5.9	1.6	[41,129]
Poly(3-hydroxybutyrate)	*Alcaligenes eutrophus*	Glucose as CS	Anion exchange resin	Packed-bed reactor (C)	74	0.04	n.a.	[130]
Carotene	*Blakeslea trispora*	Glucose and corn steep liquor as CS	Polypropylene disks	Rotary biofilm reactor (C)	144	2.4 × 10^−3^	6	[48]

^a^ batch duration; ^b^ repeated-batch or fed-batch mode; ^c^ Productivity increment corresponds to the productivity ratio between biofilms and suspended cell processes. When productivity increment is not reported, it was calculated as the ratio between the maximum productivity obtained with biofilms and the maximum productivity obtained with planktonic cultures. Abbreviations: B, batch culture; C, continuous feeding; FB, fed-batch culture; SC, semi-continuous feeding; CS, carbon source; YE, yeast extract; NS, nitrogen source; BS, biotransformation substrate; PCS, plastic composite support; n.a., not applicable.

## 3. Recombinant Proteins

**Figure 2:**
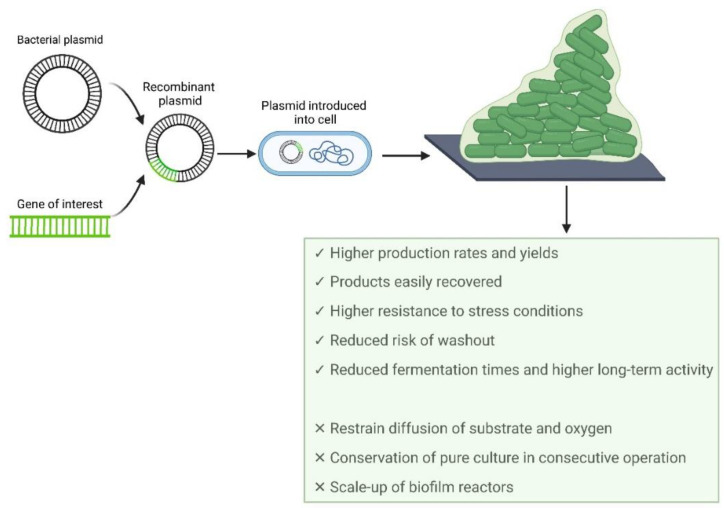
Production of recombinant proteins in biofilms: advantages and limitations.

Recombinant proteins (RPs) are a type of proteins obtained by the isolation and engineering of the gene sequence that encodes the target protein, followed by its introduction into a selected expression vector (Figure 2) [131,132].

The production of recombinant proteins requires the selection of an expression system, which should consider transcriptional and translational issues [131,132], followed by the selection of a suitable host between bacteria, yeast, filamentous fungi, mammalian, plant, and insect cells [133,134]. Despite the variety of host cells available, the selection tends to be narrowed to a few options as the host selection should take into consideration the intrinsic protein properties, level of production, cell growth, scalability potential, regulatory issues, and production cost when moving towards the industrial scale [12,135]. However, due to the considerable differences in the physicochemical properties of proteins [136], it might be difficult to predict if a target protein will be obtained in a high amount and in an active form (for example, inclusion body formation or protein inactivity may impair the yield of the target protein) [132], often requiring the development of new strategies for optimizing the production of a recombinant protein. RPs have been used in different fields of everyday life like biotechnological, food, and medical industries (Table 7).

The production of recombinant proteins has been mainly performed in suspended cell cultures. However, some studies have revealed that biofilm reactors can be a more attractive platform for their production [146,147,148]. The insertion of a gene into a multicopy plasmid imposes an added metabolic burden on the host cell due to the metabolites and energy required for the replication of plasmid DNA and the synthesis of recombinant proteins [131,149]. In planktonic cells, these events often lead to a decrease in cellular growth and biomass yields and, consequently, a decrease in the production level of the target protein [149]. On the other hand, since cells in biofilms grow more slowly than their planktonic counterparts [150], fewer resources are channeled towards replication, reducing the metabolic burden associated with plasmid maintenance [148]. Additionally, an increase in biofilm formation was evidenced due to the presence of expression vectors in bacterial cells [146]. Since stress conditions can induce biofilm formation [151], the metabolic burden related to recombinant gene expression may stimulate biofilm formation [146] and increase the production of the target protein relative to planktonic cells [146,148,152].

The production of recombinant proteins using biofilm platforms has been scarcely studied in recent decades. It predominantly resorts to bacterial cells, such as *Escherichia coli* [148,153,154] or *Bacillus subtilis* [155], fungal cells, such as *A. niger* [140] or *A. oryzae* [141], and proteins such as β-galactosidase [156,157,158] and enhanced green fluorescent protein (eGFP) [146,147,152] (Table 8).

The production of recombinant proteins in biofilms was evaluated using different platforms: microplates [144,155], parallel-plate flow cell (PPFC) systems [156,157,158], and a modified Robbins device [146,153]. Microplates are regularly used for screening assays as they are easy to handle, high-throughput platforms, and can be used in static or controlled shaking conditions [159,160]. PPFC systems enable in situ and real-time visualization of cell adhesion and biofilm formation, and require a lower medium volume when compared to modified Robbins devices; however, PPFCs have lower throughput when compared to microplates and modified Robbins devices [161]. The Robbins device was first developed to monitor biofilm formation in water systems, and since then, several modifications have been introduced, wherein some used a custom-made and semi-circular flow cell with a set of characterized hydrodynamic features [160].

Recombinant protein synthesis in biofilms was first described in bacterial biofilms in 1992 by Huang et al. [156]. They tested the production of β-galactosidase in the *E. coli* DH5α strain using a PPFC system. The production of β-galactosidase was only quantifiable when isopropyl β-D-1-thiogalactopyranoside (IPTG) induction was performed, with the maximum production being obtained 24 h after induction with yields of 0.08, 0.1, and 0.12 pg·cell^−1^ for IPTG concentrations of 0.17, 0.34, and 0.51 mM, respectively [156]. Huang et al. [157] continued to study the production of β-galactosidase with another plasmid using the same host and cultivation and induction conditions. This study revealed that β-galactosidase production reached its peak for 0.17 and 0.34 mM IPTG with 0.027 and 0.036 pg·cell^−1^, respectively, after 36 h of induction, and 0.050 pg·cell^−1^ for 0.51 mM IPTG after 48 h of induction. Moreover, β-galactosidase mRNA synthesis rates increased 4-fold under 0.17 mM IPTG, and almost 12-fold under 0.34 and 0.51 mM IPTG after 36 h of induction. Nevertheless, the production of β-galactosidase did not follow the same ratio of mRNA synthesis rate, suggesting that mRNA was less stable at higher expression levels [157].

**Table 8:** Synopsis of the published work on the production of recombinant proteins in bacterial biofilms.

Recombinant Protein	Host	Cultivation Conditions							Production Levels	Productivity Increment ^c^	Ref.
		Reactor	Surface Material	Culture Medium	Temp.(°C)	Hydrodynamics	Time (Days)	Induction			
β-galactosidase	*Escherichia coli* DH5α (pMJR1750)	PPFC	Glass	M9 minimal	37	Laminar flow (Re = 20)	4–5	IPTG (0.17–0.51 mM)	0.08–0.12 pg·cell^−1^	0.25	[156]
	*Escherichia coli* DH5α (pTKW106)								0.027–0.050 pg·cell^−1^	n.a.	[157]
eGFP	*Escherichia coli* ATCC 33456	PPFC	Glass	LB	37	Laminar flow (Re = 32)	6	-	0.01–0.16 g·L^−1^	n.d.	[148]
	*Escherichia coli* JM109(DE3)	Flow cell	PVC	Nutrient medium ^a^	30	Turbulent flow (Re = 4600)	12	-	5.8 fg·cell^−1^	30	[146]
				DM and LB			12	-	5.7–12 fg·cell^−1^	10	[154]
				LB			11	IPTG (2 mM)	17 fg·cell^−1^	n.a.	[147]
				LB and M9ZB			10	-	1.51–15.96 fg·cell^−1^	4	[162]
				TB		Transient flow (Re = 2300) and Turbulent flow (Re = 4600)	7	-	8.8–21.5 fg·cell^−1^	4	[163]
D-Amino acid oxidase	*Escherichia coli* TOP10	Static and shaken reactors	-	HSG4	30	Static conditions	7	IPTG (0.1 mM)	1.2 U·g^−1^	n.a.	[164]
			Cellulose nanofibers			170 rpm			2.1 U·g^−1^	n.a.	
Iturin A	*Bacillus subtilis*	24-well plates	-	LB	28	Static conditions	6	-	0.6 g·L^−1^	n.a.	[155]
mCherry, EgTrp and EgA31 (part of fusion proteins)	*Bacillus subtilis*	Well plates with a 22 mm^2^ surface area and agar plates	-	MSgg	30	Static conditions	3	-	n.d.	n.d.	[144]
GFP (as part of the GLA-GFP fusion protein)	*Aspergillus niger*	SFB and RFB reactor	Cotton cloth attached to a stainless-steel cylinder	Modified Vogel’s medium	25	Static conditions 100, 400, and 600 rpm	33–34	-	0.1 g·L^−1^ 0.8 g·L^−1^	n.a.	[140]
GFP (as part of the GLA-GFP fusion protein)	*Aspergillus oryzae*	BfR fungal reactor	Stainless steel packing	Complex medium ^b^	30	n.d.	3	-	n.a.	n.d.	[141]

^a^ Nutrient medium composed of 0.55 g·L^−1^ glucose, 0.25 g·L^−1^ peptone, 0.125 g·L^−1^ yeast extract, and phosphate buffer (0.188 g L^−1^ KH_2_PO_4_ and 0.26 g L^−1^ Na_2_HPO_4_), pH 7.0; ^b^ Complex medium composed of 5 g·L^−1^ soluble starch, 5 g·L^−1^ casein peptone, and 5 g·L^−1^ yeast extract; ^c^ Productivity increment corresponds to the productivity ratio between biofilms and suspended cell processes. When productivity increment is not reported, it was calculated as the ratio between the maximum productivity obtained with biofilms and the maximum productivity obtained with planktonic cultures; Abbreviations: Temp., temperature; PPFC, parallel-plate flow cell; LB, Lysogeny broth; DM, Diluted medium; Re, Reynolds number; IPTG, isopropyl β-D-1-thiogalactopyranoside; PVC, polyvinyl chloride; SFB, static fibrous bed; RFB, rotating fibrous bed; BfR, biofilm reactor: n.a., not applicable; n.d., not described. Units: pg·cell^−1^, picogram of protein per cell; fg·cell^−1^, femtogram of protein per cell; U, activity unit.

In 2007, O’Connell et al. [148] investigated the production of eGFP in a biofilm system. An *E. coli* strain harboring a pEGFP plasmid was investigated in a PPFC reactor and the authors studied the impact of ampicillin concentration on cell fluorescence, revealing that low antibiotic concentrations (between 33 and 100 ppm) lead to 60% of strongly eGFP-producing cells [148]. Further, the results revealed that the biofilm environment enhanced plasmid maintenance and heterologous protein production when compared to planktonic cells, in contrast to what was previously described by Huang et al. [156,157].

Since 2016, Gomes and collaborators have been studying the eGFP production in *E. coli* JM109(DE3) strain, both in planktonic and biofilm cells [12,146,147,152,153,154,162,163]. All studies were performed in a modified Robbins device with controlled temperature (30 °C) and hydrodynamic conditions (turbulent flow, Reynolds number of 4600, and shear stress of 0.3 Pa) throughout the assays, except for Soares et al. [163] on which turbulent and transient flow were compared. Initially, Gomes et al. [146] compared eGFP-specific production in biofilms versus planktonic cells. Experiments revealed that specific eGFP production in biofilms was about 30-fold higher than in the planktonic state, even without optimization of cultivation conditions (5.8 and 0.18 fg·cell^−1^ in biofilm and planktonic state, respectively). Afterward, Gomes et al. [154] compared eGFP production by using two different culture media (Lysogeny Broth (LB) and Diluted Medium (DM)) combined with different antibiotic concentrations (20 and 30 µg·mL^−1^ kanamycin). LB medium (composed of 10 g·L^−1^ tryptone and 5 g·L^−1^ yeast extract) has a substantial amount of carbon and nitrogen and is a medium regularly used for the expression of recombinant proteins [165]; the DM medium (composed of 0.55 g·L^−1^ glucose, 0.25 g·L^−1^ peptone, and 0.125 g·L^−1^ yeast extract) was described as a suitable medium for biofilm development [166]. The eGFP expression was higher in LB supplemented with 20 µg·mL^−1^ kanamycin with a specific production of 12 fg·cell^−1^, in opposition to 5.7 and 6.2 fg·cell^−1^ obtained with DM containing 20 and 30 µg·mL^−1^ kanamycin, respectively. Gomes et al. [154] concluded that eGFP production was higher in the LB medium and that the antibiotic concentration had no effect on the expression of eGFP. Subsequently, Gomes et al. [153] determined a set of techniques that could be performed to monitor and quantify fluorescent recombinant protein expression in biofilm cells. This study used LB medium and revealed that the biofilm population became increasingly heterogeneous during the assay, which corroborates O’Connell’s results [148]. Concerning the special distribution, eGFP-expressing cells were mostly located in the external layers of the biofilm [153]. Gomes et al. [147] also investigated the eGFP protein expression in non-induced and induced biofilms, resorting to chemical induction with IPTG at a final concentration of 2 mM. The experiment revealed that eGFP levels remained constant in the induced biofilm culture over the operation time, with a specific concentration of around 17 fg·cell^−1^, whereas in the non-induced biofilms, the eGFP production decreased by about 31%. Subsequently, Soares et al. [162] investigated the influence of nutrient conditions on recombinant protein production in biofilms comparing LB and M9ZB media. M9ZB favored biofilm development, but it had an inhibitory effect on eGFP expression, possibly due to the presence of glucose in medium composition. On the other hand, LB medium favored the number of eGFP-expressing cells and eGFP yield, probably due to the higher nitrogen content compared to M9ZB. Recently, Soares et al. [163] investigated the influence of hydrodynamics on biofilm formation and eGFP expression using Terrific Broth (TB) medium. They compared a transient flow regime (Re = 2300) with a turbulent flow regime (Re = 4600), revealing that higher biofilm eGFP production was obtained at the higher flow rate with a maximum eGFP production of 21.5 fg·cell^−1^ (2.5-fold more than under transient flow conditions).

Although GFP protein production has been mainly studied in bacterial biofilms, some research has been performed with fungal biofilms using GFP as a fusion protein. In 2005, Talabardon et al. [140] studied a recombinant *A. niger* strain containing a gene that encodes the glucoamylase-GFP (GLA-GFP) fusion protein to study the glucoamylase and GFP protein production in both suspension and immobilized biofilm cells. This study compared a static fibrous bed (SFB) and a rotating fibrous bed (RFB) under different hydrodynamics conditions and found that the RFB biofilm was able to produce 0.8 g·L^−1^ of glucoamylase and GFP, about six times more than in an SFB reactor and ten times more than in suspended cells.

Zune et al. [141] attempted the production of the GLA-GFP fusion protein in *A. oryzae* using a biofilm reactor with a stainless steel packing, whereas one bioreactor was fully immersed in the liquid medium and the other had a periodic immersion of the biofilm. The results revealed that the GFP fluorescence was similar in suspended cell cultures and biofilm reactors under high shear stress conditions. The production of the fusion protein in the two different configurations of the biofilm reactor was evaluated, revealing that both achieved similar yield values.

D-Amino acid oxidase (DAAO) is a native protein from *Rhodosporidium toruloides* and its production was performed in *E. coli* TOP10 [164]. The experiment compared static and shaken cultivation after IPTG induction, showing that DAAO protein production was nearly 2-fold higher in shaken conditions compared with static conditions.

Although most recombinant protein production in biofilms is performed in Gram-negative bacteria such as *E. coli*, some studies used Gram-positive bacteria such as *B. subtilis* to produce iturin A [155] and the fusion protein TasA [144]. Rahman et al. [155] used the *B. subtilis* 168 strain for the production of iturin A in biofilms at different temperatures, with the best production (0.6 g·L^−1^) being obtained at 28 °C. Vogt et al. [144] also used *B. subtilis* biofilms and engineered a fusion protein of TasA with the red fluorescent protein mCherry, showing that the fusion TasA-mCherry was homogeneously and abundantly distributed within the biofilm. In the same work, the production of TasA with *Echinococus granulosus* antigenic peptides (paramyosin and tropomyosin) was performed, indicating that antigens could be expressed in the biofilm state and were located in the biofilm matrix [144].

## 4. Overall Advantages and Limitations of Productive Biofilms

Biofilm reactors exhibit good operational stability with the possibility of long operation periods, increased tolerance to toxic substrates and products, robustness of the immobilized cells towards fluctuating process conditions, and high cell densities, increasing the volumetric productivity rates of several products even on dilute feed streams. On the other hand, productive biofilms may face limited oxygen and substrate diffusion and may be prone to contamination in consecutive operations. Despite these limitations, biofilm reactors have a high potential to be used in biotechnology/biotransformation processes.

Biofilm processes have been a recurring choice to produce bulk chemicals with a low ecological footprint, employing agro-wastes and biorefinery residues for their bio-conversion into valuable chemical and pharmaceutic compounds to meet economic process sustainability. In this sense, productive biofilms could have a huge potential for application in diverse areas, such as in the production of chemicals, biofuels, food additives, and bioactive compounds. Regarding the production of chemicals, to the best of our knowledge, this has only been reported at a bench and pilot scale, while the production of recombinant proteins in biofilm platforms is in its initial stage.

## 5. Future Directions

Due to the advantages of biofilm platforms over suspended cell cultures, the biotechnology industry should consider the implementation of large-scale biofilm reactors. The main advances are likely to come from the continuous development and optimization of support materials, bioreactor configurations and operating conditions, the creation of in situ biofilm monitoring strategies, and the development of suitable biofilm reactor scale-up criteria and product recovery systems. Complementary strategies such as genetic engineering of the producing microorganisms can also increase biofilm formation and even specific production rates. However, it is necessary to consider that the behavior of biofilm cultures can be hard to predict, and the lack of biofilm reproducibility can be an obstacle to its industrial application. Consequently, a study on parameters for scale-up should be performed, such as culture conditions, mass and heat transfer constraints, kinetics, and production modeling.

## Figures and Tables

**Table 7:** Recombinant proteins produced by biotechnological processes.

Protein	Application	Reference
GFP	Biotechnology Gene reporter	[137,138,139]
	Fusion tag	[140,141]
β-galactosidase	Food industry Hydrolyzation of milk products	[142,143]
	Production of galacto-oligosaccharides	[142]
mCherry	Biotechnology Gene reporter	[137]
	Fusion tag	[144]
Insulin (humulin, humalog)	Therapeutic (diabetes)	[145]
Somatropin	Therapeutic (growth)	[145]

## Data Availability

Not applicable.
